# Housing First Combined with Suicide Treatment Education and Prevention (HOME + STEP): study protocol for a randomized controlled trial

**DOI:** 10.1186/s12889-021-11133-9

**Published:** 2021-06-12

**Authors:** Laura Chavez, Kelly Kelleher, Alicia Bunger, Brittany Brackenoff, Ruri Famelia, Jodi Ford, Xin Feng, Allen Mallory, Jared Martin, Arielle Sheftall, Laura Walsh, Tansel Yilmazer, Natasha Slesnick

**Affiliations:** 1 Center for Child Health Equity and Outcomes Research, Nationwide Children’s Hospital, 700 Children’s Drive, Columbus, OH 43205 USA; 2 College of Social Work, The Ohio State University, 1947 N. College Road, Columbus, OH 43210 USA; 3 College of Education and Human Ecology, Department of Human Sciences, The Ohio State University, 1787 Neil Ave, Columbus, OH 43210 USA; 4 College of Nursing, The Ohio State University, 1585 Neil Avenue, Columbus, OH 43210 USA

**Keywords:** Housing, Homelessness, Young adults, Suicide, Opioid use

## Abstract

**Background:**

Youth experiencing homelessness are at high risk for suicide, yet few studies have evaluated risk reduction interventions targeting suicidal ideation in this vulnerable population. A comprehensive approach to risk-reduction is needed that addresses basic needs and provides targeted interventions for those at highest risk. The protocol described builds on the design of the first randomized trial of Housing First (HF) for homeless youth. The primary objective is to determine whether housing combined with supportive services that include suicide screening and targeted psychotherapy (Cognitive Therapy for Suicide Prevention) is effective for reducing suicidal ideation and other secondary outcomes (depression and suicide attempts). Additionally, we will explore mediators of the treatment effect (housing stability and substance use) and determinants of implementation.

**Methods:**

Youth recruited to the HF trial will be randomized to HF + supportive services (*n* = 120), or supportive services alone (*n* = 120). The “Suicide Treatment Education and Prevention” (STEP) protocol will additionally screen youth in both arms at baseline and 3 months for suicidal ideation (SSI-W). Those who screen as moderate risk for suicide (SSI-W ≥ 10) will be offered CTSP, which includes up to 9 sessions over the first 6 months following enrollment. CTSP will be delivered in one-on-one sessions by a trained advocate. Research assessments will be collected to assess outcomes (including suicidal ideation) at baseline, 3, 6, 9 and 12 months. Qualitative interviews with subjects receiving CTSP and other stakeholders will explore implementation determinants.

**Discussion:**

The study will fill an important gap in the literature about the added benefit of HF combined with supportive services including suicide screening and treatment for reducing suicidal ideation in homeless youth. With the urgent need to address both homelessness and suicide risk, evidence is needed about services that can be integrated into delivery settings for youth experiencing homelessness.

**Trial registration:**

NCT04135703. Date of registration: October 23, 2019.

## Background

Deaths from suicide among youth under age 24 years are increasing in the United States and suicide was the second leading cause of death in 2019 [1]. The COVID-19 pandemic has only increased the dire risk for suicide in young adults, with elevated reports of suicidal ideation in nationally representative U.S. samples in 2020 compared to 2018 (10.7% vs 4.3%) [2, 3]. Some populations of youth are at higher risk for suicide than others, particularly youth experiencing homelessness who have high rates of death from suicide in longitudinal studies [4, 5]. An estimated 1 in 10 youth ages 18–25 (3.5 million) experience homelessness in a given year in the U.S. [6, 7]. Homeless youth, a term often used to describe homeless adolescents and young adults under age 25 [8], are a vulnerable population who often experience trauma both before and after becoming homeless [9–11], as well as significant mental health and substance use comorbidity. The lived experiences of homeless youth combined with a high prevalence of substance use disorders put them at increased risk for suicidal ideation and attempts [12–14]. Studies estimate approximately 40% of homeless youth have attempted suicide at least once in their lifetime [11, 12], much higher than the 1.9% of young adults in the U.S. general population who reported suicide attempts in a 2018 household-based survey [15].

This randomized trial, entitled **Housing First Combined with Suicide Treatment Education and Prevention (HOME + STEP)**, is a secondary protocol that was funded as a competitive revision to the original trial with an additional focus on suicide prevention for both arms of the trial [16]. The parent study is a randomized controlled trial of housing first (HF) combined with opioid and related risk preventive interventions (*n* = 120), relative to opioid and related risk preventive interventions alone (*n* = 120). The primary outcomes are 1) time to opioid use disorder (OUD) and 2) percent of days of opioid use at follow-up (3, 6, 9, 12 months). In the present protocol description, we describe the design of the secondary study, including how the suicide prevention services will be delivered and how the study will answer important questions regarding the added benefit of housing first (HF) for addressing suicide risk in a high-risk population of youth.

### Evidence for suicide prevention and recommendations

The U.S. Surgeon General recently released a call to action for fully implementing the National Strategy for Suicide Prevention, which was last updated in 2012 [17, 18]. Although progress has been made toward some goals, more work is needed. Six action items were highlighted by the U.S. Surgeon General [17]. Among these, the call to address upstream risk factors for suicide has salience for homeless youth. Some examples to address these risks for homeless youth include increasing social connection, strengthening economic supports, and engaging underserved groups in preventive services [17]. As noted by the Centers for Disease Control and Prevention’s technical guidance for implementation of the National Strategies [19], a comprehensive approach to addressing these goals is needed and should include identifying those at higher risk for suicide, delivering evidence-base preventive interventions such as psychotherapy, and efforts to increase financial stability. However, though homeless populations are recognized as vulnerable to suicide in these national reports, research identifying effective interventions to reduce their risk for suicide and ways of integrating interventions into comprehensive strategies that also address financial instability is lacking.

Cognitive Therapy for Suicide Prevention (CTSP) is an evidence-based intervention that shows promise in adults and youth with recent suicide attempts treated in hospital settings [20, 21]. A recently completed trial of CTSP builds the evidence-base for adaptations of effective interventions that can be delivered to address suicide risk among homeless youth. Among 18–24 year old homeless youth with suicidal ideation at high risk for suicide (*n* = 150), suicidal ideation declined faster among youth randomized to CTSP relative to those in usual care [22, 23]. Importantly, the trial of CTSP among homeless youth demonstrated effectiveness and was delivered at a homeless youth drop-in center by the center’s staff therapists. Thus, this adaptation of CTSP demonstrates the potential to increase access in nontraditional settings. However, CTSP did not directly address housing instability experienced by homeless youth and most remained unhoused by the end of the study. Furthermore, low rates of participation for more than two or three visits hindered the fidelity of the treatment. It is unknown whether the addition of supportive housing to CTSP could further improve suicide-related outcomes by reducing stress, decreasing trauma, and allowing for more effective service delivery.

### Potential of housing First (HF) for addressing suicide risk

Providing housing and advocacy support increases financial stability and could have benefits for reducing suicidal ideation. The HF model is predicated on providing housing without preconditions of sobriety [24, 25]. Relative to community services as usual, prior controlled trials of HF in adults demonstrates efficacy for improving housing stability and reducing substance use [25] [26]. Following exposure to HF, suicidal ideation was significantly reduced in two prior studies [27, 28], one of which was a randomized trial of HF [28]. However, among chronically homeless adults with severe mental illness, suicidal ideation was not significantly reduced when compared to a community services control group [28]. These results suggest targeted suicide prevention may be needed for individuals with severe suicidal ideation who are at highest risk for suicide. There is also a critical need to study both effectiveness and implementation of evidence-based suicide prevention strategies to increase research translation [29].

The increases in housing stability afforded through HF could impact how youth interact with service providers and has implications for implementation of suicide prevention interventions. Maintaining contact with youth who are experiencing homelessness is a known challenge for service providers [30]. However, when youth have a stable residence, they may be better able to participate in therapy sessions and barriers to maintaining contact with youth could be alleviated. In addition, youth may engage more fully in offered services when they aren’t focused on meeting immediate needs such as finding safe shelter. Exploring the determinants of implementing CTSP and possible differences based on housing status from both the perspectives of youth and stakeholders can inform future dissemination of suicide prevention among homeless youth. For example, differences may highlight variable implementation strategies needed to deliver suicide prevention based on youth receiving supportive housing services.

The present study will formally test a comprehensive system of care that addresses basic needs, while also offering higher levels of targeted intervention to those at highest risk of suicide. The HOME + Suicide Treatment Education and Prevention (STEP) will deliver comprehensive suicide screening for all enrolled youth and offer CTSP for those at highest risk in both arms of the study. The primary objective is to determine the effectiveness of HOME + STEP, compared to STEP alone for reducing primary (suicidal ideation) and secondary (depression symptoms, suicide attempt) outcomes. We hypothesize that youth in the HOME + STEP arm will show greater improvement in outcomes over time, relative to the STEP alone arm. Secondary objectives include 1) exploring potential mediators of the treatment effect (substance use, housing stability, hopelessness, and stress coping); and 2) evaluating the treatment effect in a high-risk subgroup identified based on screening protocol, and 3) exploring determinants of implementation of CTSP across both conditions. The study will explore the implementation determinants of delivering CTSP to high-risk youth in both groups using mixed methods approach to inform future dissemination and implementation of suicide prevention services. We hypothesize youth who are randomized to receive HF (HOME + STEP) will participate more fully in CTSP.

## Methods/design

### Study design

The “HOME Suicide Treatment Education and Prevention Study (HOME + STEP)” study builds on the parent study design and data collection schedule [16]. The HOME + STEP revision uses the full proposed sample from the parent study (*n* = 240) and adds supplemental measures to assess suicidal ideation and other important measures (e.g., suicide attempts, hopelessness). The present study delivers suicide screening and prevention to all youth, as well as CTSP for those at highest risk in both arms of the study based on screening procedures described below. The randomized design of the HOME study (*n* = 240) allows for testing the primary objective of comparing the effectiveness of HOME + STEP (*n* = 120), compared to STEP alone (*n* = 120) for reducing primary (suicidal ideation) and secondary (depression symptoms, suicide attempts). Upon completion of the baseline assessment interview, youths will be randomly assigned to the HF intervention condition using a computerized urn randomization program. All youth will be screened at baseline and 3 months using the Scale for Suicide Ideation – Worst Point (SSI-W) [31]. Those who receive a score of 10 or higher, indicating moderate suicide risk, or report a past-year suicide attempt will be eligible for 10 sessions of CTSP, provided by research staff. Based on our pilot project (UG3), we expect approximately *N* = 100 youth will meet the criteria for receiving CTSP, and these youth should be nearly equally distributed in HOME + STEP (*N* = 50) and STEP alone (*N* = 50).

### Participants

Consistent with the main inclusion criteria for the parent trial, youth will be eligible if they meet the following criteria: 1) age 18 to 24 years; 2) meet the criteria for homelessness, as defined by the federal McKinney-Vento Act (2002) as “lacking a fixed, regular, stable, and adequate nighttime residence” and includes “living in a publicly or privately operated shelter designed to provide temporary living accommodations, or a public or private place not designed for, or ordinarily used as, regular sleeping accommodations for human beings;” [32] and 3) youth fail to meet *Diagnostic and Statistical Manual of Mental Disorders, 5th edition* (DSM-5) criteria for Opioid Use Disorder as assessed by the Structured Clinical Interview for DSM-5 Disords (SCID) [33]. Youth will qualify for CTSP if the meet either of the following: 1) Youth receives a score of 10 or higher on the Scale for Suicide Ideation – Worst Point (SSI-W) [34]; or 2) any self-reported suicide attempt in the past year at baseline, or 3) any report of a suicide attempt from baseline through the first 6 months of enrollment. Thus, youth who did not initially screen into STEP at baseline will have an additional opportunity to screen into STEP if they meet the above criteria before the 6-month follow-up assessment. Severe suicide ideation is defined as scoring 16 or higher on SSI-W [31]. Beck et al. (1999) reported that clients who scored 16 or higher on the SSI-W had 14 times higher odds to die by suicide. However, given our prevention goal, we will engage those with a score of 10 or higher so that moderate risk youth are engaged as well. Similar to a study by Stanley and colleagues (2009), suicidal ideation will be considered recent if it occurred in the prior 90 days.

### Recruitment protocol

**Fig. 1:**
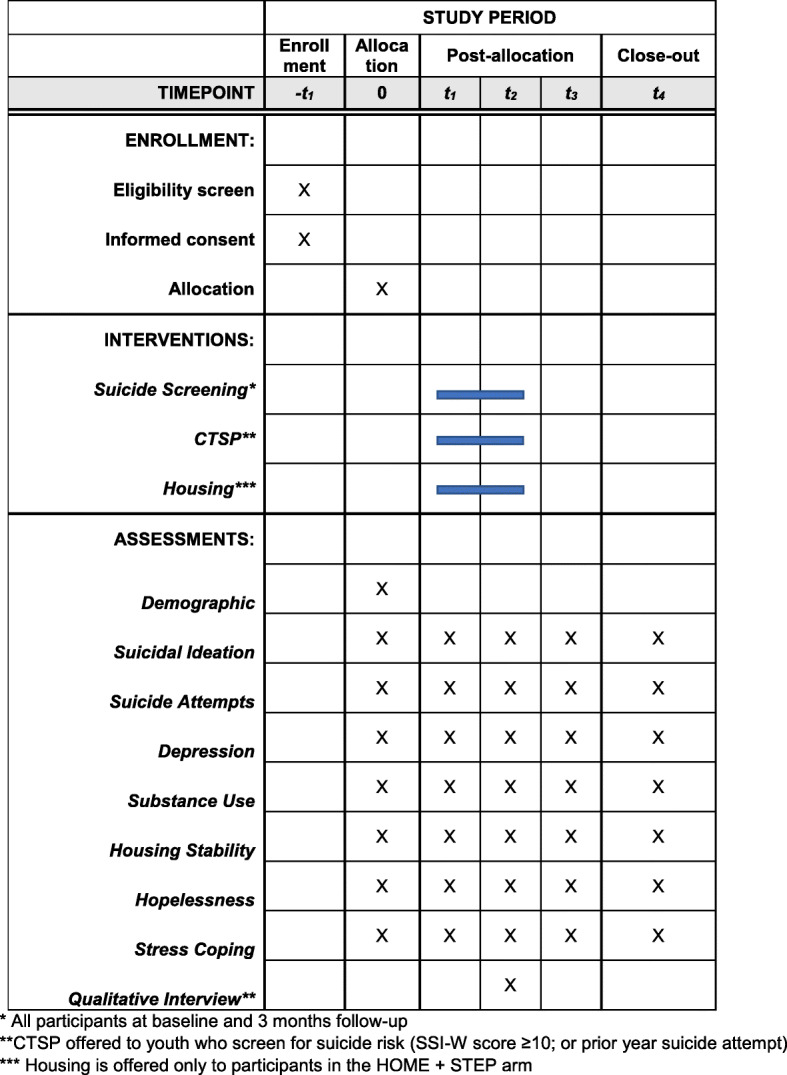
Schedule of enrollment, interventions, and assessments. * All participants at baseline and 3 months follow-up. **CTSP offered to youth who screen for suicide risk (SSI-W score ≥ 10; or prior year suicide attempt). *** Housing is offered only to participants in the HOME + STEP arm

As under the parent study, youth who attend a homeless youth drop-in center in a large Midwestern City will be recruited to participate in the study. The protocol for the parent study has been described previously [16]. Briefly, research staff will engage with youth who attend the drop-in center by briefly describing the study and conducing a brief screener (Fig. 1). Youth who are eligible and interested in the study will be further assessed to confirm they meet eligibility criteria and among those who are eligible, research staff will obtain informed consent. Study recruitment and screening procedures will be conducted in a research space present at the drop-in center.

### Interventions

#### Suicide screening

Identifying those at risk of suicide is a core objective in national strategies to prevent suicide [18, 19]. Unfortunately, youth who experience homelessness have limited access to medical care or mental health services and are thus unlikely to be screened for suicide in traditional settings. More efforts are needed to identify homeless youth at risk for suicide and link them to appropriate resources. The study will use the SSI-W to assess suicide risk at baseline and 3-month follow-up assessments, as well as past-year attempt at baseline (Fig. 1). In addition, if suicide attempts are reported to advocates at any time during the first 6 months of enrollment youth will be offered CTSP. Protocol to address imminent suicide risk through established emergency procedures (see below) will be followed for any individuals who screen for suicide risk. Those judged not at imminent risk will remain in the project and repeat screening; those with imminent risk will follow emergency procedures for hospitalization when appropriate and be referred to CTSP.

#### Cognitive therapy for suicide prevention (CTSP) for high risk youth

CTSP is based upon the theoretical assumption that people’s thoughts and interpretation of life events determines their emotional and behavioral responses [35]. Therefore, maladaptive cognitions associated with suicidal ideation are the primary focus of the treatment. The intervention protocol was developed to identify cognitive processes relevant to suicidal acts and targets vulnerability factors including: hopelessness, social isolation, poor problem solving, and impaired impulse control. The treatment is designed as a 10-session protocol including weekly or bi-weekly meetings (Table 1) [20, 21, 35]. All subjects with severe suicidal ideation at baseline will receive the therapy from a trained research staff member. The therapy will be delivered by phone or in-person, depending on participant preference and safety precautions for COVID-19. If in person, sessions will occur in a private room set aside for research at the youth drop-in center or, alternatively, in a private room set aside for counseling at a transitional housing unit (if participant randomized to HF). However, youth will be offered up to 9 additional maintenance sessions over a six-month period, tapered in frequency, consistent with treatment offered in prior studies [20, 21]. To increase treatment participation, youth will receive a $5 food gift card (e.g. McDonald’s, Burger King, Wendy’s, or other food gift card) for every CTSP session they attend.

**Table 1:** Techniques and Session Structure for Cognitive Therapy for Suicide Prevention (CTSP)

Session 1–2	Rapport Building with Therapist, Review Safety Planning
Session 3	Develop a hope kit, which includes reasons to live, such as memories, letters, pictures or other reminders of significant relationships with others.
Session 4–6	Build support network with other service providers, as well as social support. Identify the maladaptive thoughts and core beliefs (e.g. view of self or/and others), and help the client develop alternative ways of thinking and behaving.
Session 7–10	Develop coping strategies with suicidal crises, such as relaxation, destruction, and intense physical sensations (e.g. holding ice).
Additional Sessions	Provide continued support to reinforce therapy objectives and relapse prevention.

#### CTSP training and supervision

The research staff will receive a 3-day training following the manualized “Cognitive Therapy for Suicidal Patients” [35]. Staff who are trained to serve as youth advocates will receive this in addition to other training for the parent study [16]. All sessions will be digitally recorded, and supervision will be led by the senior author (Dr. Slesnick). The recorded sessions will be rated for quality prior to supervision sessions which occur biweekly.

#### Emergency procedures for current suicidal ideation

During research assessments or CTSP therapy sessions, if youth reports any thoughts of suicide at any point in the interview or self-report responses, the assessment of imminent risk will be completed, including 1) whether a plan is in place, 2) whether the means to carry out the plan are available, and 3) whether there is intent to carry out the plan, as well as reasons for wanting to harm themselves. Research staff will then call the senior author to review the youth’s responses and discuss next steps. If imminent risk is determined, the client is asked to accompany the staff member to seek immediate services at a mental health crisis intervention, stabilization, and assessment center. The crisis center is staffed by skilled physicians, nurses, psychologists, social workers, counselors, and technicians and is open 24 h a day. Crisis center staff will coordinate treatment, such as hospitalization, through local agencies when necessary. Services are available to anyone, regardless of the ability to pay. If the subject refuses to accompany the staff to the crisis center, then the police are called to accompany the youth.

### Ethics and human subjects review

The study protocol has been reviewed and approved by the Ohio State University’s (OSU) Institutional Review Board. In addition, a Data Safety Monitoring Board that includes three members experienced in clinical trials, biostatistics, and conducting research among homeless youth populations meets twice per year to review study progress and adverse events. Serious adverse events that are unanticipated and may be related to study procedures will be reported to the OSU IRB and sponsor (NIDA) within 48 h of learning of the event. Adverse events that are unexpected and potentially related to the study, but not meeting the definition of a serious adverse event will be reported to the IRB within 10 days. The OSU IRB reviews the adverse event report and determines if the event is a result of study procedures. If the event is considered a direct result of study procedures, the MPIs and the data safety monitoring board (DSMB) will meet within 48 h to discuss whether protocol modifications are needed. The trial will be stopped if any intervention is found to cause harm to participants.

### Data collection and measures

Trained research staff will conduct in-person interviews at baseline, 3, 6, 9, and 12 months (Fig. 1). Subjects will be compensated for completing study measures at $50 per interview. Those who participate in the qualitative interviews will be compensated $20. Subjects will also be provided $5 incentives to participate in advocacy sessions or CTSP sessions. Under the parent protocol, the research staff receive a training on research assessments, youth engagement, and tracking procedures. Weekly supervision is used to brainstorm engagement and tracking strategies of clients. To maintain contact with enrolled youth and increase retention in the study, extensive locator information will be collected at the baseline assessment and all follow-up assessments (e.g. collateral contacts or frequent hangouts) to prevent attrition. Further, quality control of assessment procedures (double data entry and verification) will be reviewed during these meetings. Assessors will be blinded to treatment condition. Paper copies of study data will be secured in a locked file cabinet in locked rooms within the drop-in center and/or university office. Electronic data will be maintained on a password protected electronic files on secure servers behind the university firewall. The final trial results will be reported in peer-reviewed publications but will not be shared directly with trial participants. A de-identified dataset will be shared with the sponsor and made publicly available.

#### Primary outcome

The primary outcome for this study is *suicidal ideation*. Suicidal ideation will be assessed with the 19-item Scale for Suicide Ideation – Worst (SSI-W) [34], an interviewer-administered rating scale. The SSI-W is also used to assess youth’s eligibility for CTSP at baseline or over the 6-month period (as described above). The SSI-W will assess suicidality over the past 90 days, as in previous studies. The SSI-W was developed to identify the intensity of the most severe suicidal ideation experienced by the person. The scale measures the intensity of patients’ attitudes, behaviors, and plans to die by suicide during the time period [34]. Interviewers instruct respondents to recall the time when they had their most intense thoughts and desire to die by suicide. Respondents are then asked to keep this time in mind while the interviewer rates responses regarding how suicidal they were at that this specific time. The SSI-W has moderately high internal consistency (Cronbach alpha = .88) and validity, and associations with other measures of suicidal ideation including the SSI, single items from the Beck Depression Inventory, and Hamilton Rating Scale for Depression [31].

#### Secondary outcomes

*Suicide attempts* will be assessed with a single self-report item similar to that used by the Ask Suicide-Screening Questions (ASQ) [36], that has been used in prior studies among homeless youth and is modified to ask about past 12 months at baseline, and past 3 months for follow-up assessments. *Depression symptoms* will be assessed with the Beck Depression Inventory (BDI), which is collected as a secondary outcome under the parent HOME trial.

#### Potential mediators

If the HOME + STEP intervention is shown to be effective for improving outcomes, the study will explore primary and secondary mediators of the treatment effect. *Substance use* and *housing stability*, which have both been shown to improve with exposure to HF in adult samples [25, 26, 37], will be explored as primary mediators. The Form 90 will measure substance use (alcohol or other drug, excluding tobacco) and housing stability [38, 39], which is consistent with the parent trial and other prior studies among homeless youth [16, 40]. For these variables, percent days of any substance use and percent days house will be calculated. *Hopelessness* will be measured with the Beck Hopelessness Scale (BHS) [41], a self-report instrument that consists of 20 true-false statements designed to assess the extent of positive and negative beliefs about the future during the past week. The BHS is one of the most widely used measures of hopelessness and has demonstrated high internal reliability across diverse clinical and nonclinical populations (Kuder-Richardson reliabilities ranging from .87 to .93) [41]. The BHS has adequate one-week test-retest reliability in a psychiatric outpatient sample (r = .69) and high three-week test-retest reliability in a college student sample (r = .85) [42]. *Stress coping* will be measured with the Coping Inventory for Stressful Situations-Short Form (CISS-SFC) [43], which is collected as a secondary outcome of the parent trial and has been used in prior studies of homeless youth.

#### Qualitative interviews

To increase our understanding of the determinants of CTSP implementation including its *acceptability, appropriateness,* and *feasibility*, we will use qualitative data collection. Up to 20 youth who receive CTSP, will be interviewed. Qualitative interviews will be conducted by trained research staff. All interviews will be audio recorded and transcribed by a transcription service. Youth will be queried about their experience with CTSP at the end of the treatment period (6 months) and asked to discuss aspects of the CTSP intervention they benefited from and aspects they did not find helpful. This information will give a better understanding of factors that promote engagement among youth as well as factors that may prevent youth from engaging in the intervention. We will also interview research staff who delivered CTSP and community stakeholders (*n* = 5). Community stakeholders will include representatives from organizations serving homeless youth, such as the city’s homeless youth drop-in center leadership and other service providers. Interviews of therapists and stakeholders will include open-ended questions about what it would require to implement CTSP (e.g., limitations/barriers). In addition, specific probes will be included that focus on domains from a widely used implementation framework, the Consolidated Framework for Implementation Research (CFIR) [44].

#### Implementation process measures

CTSP treatment *fidelity* will be assessed based on the “dose” delivered using study records and therapist ratings. Study records will measure treatment attendance including number of sessions and proportion of youth completing high levels of therapy (80%). The Cognitive Therapy Rating Scale (CTRS) [45], an 11-item scale developed to assess therapist competence, will also be used to assess fidelity. Items are scored on a 7-point scale: 0 (Poor), 1 (Barely Adequate), 2 (Mediocre), 3 (Satisfactory), 4 (Good), 5 (Very Good), and 6 (Excellent). The 11 items are summed to yield a CTRS total score, ranging from 0 to 66 (≥40 considered competent). The CTRS will be completed by the senior author during supervision. CTSP ***costs*** will be measured as the advocate time for training, delivering therapy, supervision, and contacting youth for follow-up appointments or rescheduling as well as the labor, equipment, and space costs, based on an activity-based costing method and semi-structured, in-person interviews of therapists and other relevant personnel [46].

### Analytic plan

#### Missing data

The patterns of missing data will be first examined before each analysis. If data are missing at random [47], they will be estimated using full information maximum likelihood (FIML) or multiple imputations (MI) method. When data are missing completely at random (MCAR) or are missing at random (MAR), both FIML and ML produce unbiased results [48]. The main statistical program used for this study, MPlus, can handle missing data by providing FIML estimation. If the missingness cannot be explained by observed data, that is, data are Missing Not at Random (MNAR), data analysis will be conducted using the pattern mixture model framework [49].

#### Primary aim 1. The effect of the housing intervention on suicidal ideation

The impact of housing on the primary (suicidal ideation) and secondary outcomes (depressive symptoms, suicide attempts) over time will be tested between the two main treatment conditions: housing combined with risk preventive services and risk preventive services alone. For these analyses we will control for CTSP treatment attendance, which has been positively associated with treatment outcomes [50, 51]. Specifically, the primary and secondary outcomes will be analyzed using latent growth models (LGM) to estimate the trajectories of change across five time points (baseline, 3, 6, 9, and 12 months follow-up). LGM can also estimate joint trajectories with multiple outcome variables. Differences between the main treatment conditions on estimated growth parameters, including intercepts (i.e., initial status) and slopes (i.e., the rates of change), will be tested. It is expected that those assigned to the housing combined with risk preventive services group will show greater decreases in suicidal ideation and depressive symptoms, and that these improvements will maintain for a longer period of time, compared to those assigned to the prevention services alone group. We anticipate that number of participants reporting suicide attempts in the current sample will be relatively low. Thus, in the LGM analysis, we will dichotomize the measure of suicide attempts (i.e., presence vs. absence of suicide attempt) to test whether the likelihood of suicide attempt decreases faster in the combined housing and services group than in the services alone group. To increase validity of the conclusions drawn from the analysis, the number of intervention sessions and contacts with other service providers will be covariates in the LGM analyses.

#### Secondary aim 1. Testing potential behavioral change mechanisms (i.e., housing and substance use)

A series of path analysis will be conducted to test the mediation hypotheses. *Primary mediators*: we will first test whether percent of days housed and percent days of substance use assessed at the 3- or 6-month follow-up mediates the association between intervention (combined house and services vs. services alone) and outcomes at 6-month and 9−/12-month follow-up, respectively. We expect that the intervention condition with the housing component will produce greater improvements in percent day housed and substance use, which in turn will predict reduced suicide ideation, depressive symptoms, and likelihood of suicide attempt. *Secondary mediators*: we will test the effects of hopelessness and stress coping on the relationship between primary mediators and outcomes. We expect that lower housing instability and substance use at 3 months will be associated with lower hopelessness and greater stress coping at 6, which will in turn be associated with reduced suicidal ideation, depressive symptoms, and likelihood of suicide attempts at 9−/12-months. Following MacKinnon and colleagues [52], the product of the coefficient of the path from the independent variable to the mediator(s) and the coefficient of the path from the mediator(s) to the outcomes will be computed as the indirect (mediation) effect between the intervention and outcomes. The strength and significance of the mediation will be estimated using a bootstrap sampling method [53]. Similar to the primary aim, we will control for CTSP treatment attendance, number of intervention sessions, and contacts with other service providers in the analysis.

#### Secondary aim 2. Testing the intervention effect for high-risk youth (SSI-W ≥ 10) eligible for CTSP at baseline

We will only include youth at high risk of suicidality in the analyses to provide an estimation of the effect of housing on high levels of suicidality between the two groups: housing combined with services and services alone. The expected sample size is approximately 100. Repeated measures ANOVA (RMANOVA) will be conducted to examine the differences between the groups in continuous outcomes, suicidal ideation, and depressive symptoms. Specifically, a 3 (treatment condition) × 5 (time points) RMANOVA will be conducted with treatment as the between-subject factor and time as the within-subject factor, in order to test the differential change pattern over time between the two groups. For suicidal attempts (both count and dichotomous variable) generalized linear mixed models (GLMM) will be conducted. In the event that the high-risk sample would not provide sufficient power for the GLMM analysis, further exploration analysis will be conducted on the dichotomous suicidal attempts variable to examine the housing effect at the end of treatment (i.e., 6-months follow-up) and the end of the study (i.e., 12-month follow-up), using logistic regression, controlling for the baseline assessment.

#### Secondary aim 3. Explore the determinants of implementation

To understand how the determinants of CTSP implementation vary across settings, we will use a mixed-methods design. In the quantitative phase, we will examine and compare quantitative data on fidelity and costs across both study arms. Average start-up and ongoing estimated costs for all STEP-related services will be presented overall and across study groups. Qualitative interviews will be iteratively coded using a modified grounded theory approach – modified in that we will orient our initial open coding process around what Patton describes as “sensitizing concepts” including issues related to acceptability, appropriateness and feasibility [54, 55], NVivo 11 software will be used to organize and code the data. Data will be used to identify salient contextual features service delivery that need to be addressed in future implementation efforts and will provide greater explanation about why there are (or are not) differences in implementation outcomes across conditions. Results may also provide insight into which implementation strategies might be needed to deliver CTSP.

#### Statistical power analysis

The power analyses were conducted using the Monte Carlo simulation method for the Primary Aim and Secondary Aim 1 and using GPower program for Secondary Aim 2. We assumed an overall attrition rate of 15% when calculating the power. The MPI’s ongoing study testing the effect of housing support on homeless mothers reveals medium effect size in reducing depressive symptoms (*d* = .59), favoring the housing intervention over treatment as usual (TAU)/assessment only. Few studies examined the effect of housing on suicidality; a recent observational study reported a small effect size from baseline to 2-year follow-up [27]. Given the large difference across studies and that the proposed study tests housing support against an active intervention, and the differences between the two conditions may be smaller than that between housing support and TAU, we assumed small-to-medium effect sizes when conducting the power analysis. For the Primary Aim, with dichotomous predictors (contrasts between intervention conditions) that have regression coefficients of .15 (small-to-medium effect size) for the slopes of growth factors [56], a sample size of 240 can produce a power of .86 to detect the housing effect on the growth rate of outcomes. For Secondary Aim 1, the MPI’s clinical trial of housing support suggested a large effect size (*d* = 1.26) in improving housing stability and a medium effect size in reducing substance use (*d* = .49). Similarly, a meta-analysis reported large effect sizes (*d* = 1.24 on average) of Housing First approaches in increasing housing stability [57]. Thus, we assumed a medium effect size for the intervention-to-mediator paths and a small-to-medium effect size for the mediator-to-outcome paths. Following the model specification suggested by Thoemmes et al. (2010) [58], the proposed sample size could provide a power of .91 to detect mediating effects for models with one mediator and a power of .82 for the serial mediation models with two mediators. For Secondary Aim 2, assuming a small-to-medium effect size (i.e., *f* = .20), the power to detect a group difference in an RMANOVA with 5 repeated measurements is .84. For the GLMM, to achieve 80% power, the effect size of the group differences in the slope needs to be of medium size (regression coefficient of .20). A smaller than medium effect size will not provide sufficient power to detect housing effect on suicidal attempts. However, our goal is to estimate an effect size that can be used to guide sample-size estimates for a future fully-powered study.

## Discussion

The HOME + STEP study will provide information to stakeholders on the effectiveness of supportive housing combined with suicide prevention for reducing suicidal ideation and addressing other secondary outcomes in homeless youth. Programs for housing transitional age youth experiencing homelessness have been implemented in cities across the United States and many observational studies have noted improvements in housing stability and reduced drug use. At a time when the homelessness and mental health crises in the United States are converging during the COVID-19 pandemic, evidence for whether supportive housing can effectively reduce suicide risk and related outcomes is needed. Observational studies alone cannot provide stakeholders with the information needed to determine the extent to which supportive housing programs can improve upon preventive interventions alone. The financial resources needed for implementing housing interventions are great, but if health and well-being are improved this information can be used to argue for the importance of this investment.

## Data Availability

Not applicable.

## Abbreviations

ANOVA: Analysis of variance
ASQ: Ask suicide – screening questions
BDI: Beck depression inventory
BHS: Beck hopelessness scale
CFIR: Consolidated framework for implementation research
CISS-SFC: Copying inventory for stressful situations – short form
CTSP: Cognitive therapy for suicide prevention
DSM-5: Diagnostic and statistical manual of mental disorders, 5th edition
DSMB: Data safety monitoring board
FIML: Full information maximum likelihood
GLMM: Generalized linear mixed models
HF: Housing first
HOME+STEP: Housing first combined with suicide treatment education and prevention
IRB: Internal review board
LGM: Latent growth models
MAR: Missing at random
MCAR: Missing completely at random
MI: Multiple imputations
MNAR: Missing not at random
MPIs: Multiple PI
NIDA: National institute drug abuse
OSU: The Ohio state university
OSUIRB: The Ohio State university internal review board
OUD: Opioid use disorder
RMANOVA: Repeat measures ANOVA
SCID: Structured clinical interview for DSM-5 disords
SSI-W: Scale for suicide ideation – worst point
TAU: Treatment as usual
